# 

**Published:** 1868-11-15

**Authors:** 


### CONTENTS:

ORIGINAL PAPERS:

The Virulence and Specificity of Tuberculosis. Translated by Walter Hay, M.D., Associate Editor, Chicago.................. 711

Carcinoma of the Spleen. By V. R. Bridges, M. D., Matoon, Ill.. 729

Experiments on Calculi, in and out of the Body, etc. By T. Williams, M.D., Milwaukee, Wis.................................. 734

EDITORIAL:

Prof. E. S. Carr .............................................. 739

Hegeman & Co.................................................. 740

Rush Medical College Library and Reading Room................. 740

Items.......................................................... 740

BOOKS AND PAMPHLETS RECEIVED.................................. 740

OBITUARY—Walter Walker, M.D................................... 741

LOOT........................................................... 741

